# First recorded eruption of Nabro volcano, Eritrea, 2011

**DOI:** 10.1007/s00445-015-0966-3

**Published:** 2015-09-07

**Authors:** Berhe Goitom, Clive Oppenheimer, James O. S. Hammond, Raphaël Grandin, Talfan Barnie, Amy Donovan, Ghebrebrhan Ogubazghi, Ermias Yohannes, Goitom Kibrom, J- Michael Kendall, Simon A. Carn, David Fee, Christine Sealing, Derek Keir, Atalay Ayele, Jon Blundy, Joanna Hamlyn, Tim Wright, Seife Berhe

**Affiliations:** School of Earth Sciences, University of Bristol, Queens Road, Bristol, BS8 1RJ UK; Department of Earth Sciences, Eritrea Institute of Technology, PO Box 12676, Asmara, Eritrea; Department of Geography, Downing Place, Cambridge, CB2 3EN UK; Department of Earth Science and Engineering, Imperial College, London, SW7 2AZ UK; Institut de Physique du Globe de Paris, Sorbonne Paris Cité, Univ Paris Diderot CNRS, 75005 Paris, France; Laboratoire Magmas et Volcans, Université Blaise Pascal, Clermont Ferrand, France; Department of Mines, Eritrea Geological Surveys, PO Box 272, Asmara, Eritrea; Department of Geological and Mining Engineering and Sciences, Michigan Technological University, 1400 Townsend Dr, Houghton, MI 49931 USA; Wilson Infrasound Observatories, Alaska Volcano Observatory, Geophysical Institute, University of Alaska Fairbanks, Fairbanks, AK USA; National Oceanography Centre Southampton, University of Southampton, Southampton, SO14 3ZH UK; Institute of Geophysics, Space Science and Astronomy, Addis Ababa University, Addis Ababa, Ethiopia; COMET, School of Earth and Environment, University of Leeds, Leeds, UK; Global Resources Development Consultants, Asmara, Eritrea

**Keywords:** Nabro, InSAR, Seismicity, Afar, Danakil, Volcano monitoring, Satellite remote sensing

## Abstract

We present a synthesis of diverse observations of the first recorded eruption of Nabro volcano, Eritrea, which began on 12 June 2011. While no monitoring of the volcano was in effect at the time, it has been possible to reconstruct the nature and evolution of the eruption through analysis of regional seismological and infrasound data and satellite remote sensing data, supplemented by petrological analysis of erupted products and brief field surveys. The event is notable for the comparative rarity of recorded historical eruptions in the region and of caldera systems in general, for the prodigious quantity of SO_2_ emitted into the atmosphere and the significant human impacts that ensued notwithstanding the low population density of the Afar region. It is also relevant in understanding the broader magmatic and tectonic significance of the volcanic massif of which Nabro forms a part and which strikes obliquely to the principal rifting directions in the Red Sea and northern Afar. The whole-rock compositions of the erupted lavas and tephra range from trachybasaltic to trachybasaltic andesite, and crystal-hosted melt inclusions contain up to 3,000 ppm of sulphur by weight. The eruption was preceded by significant seismicity, detected by regional networks of sensors and accompanied by sustained tremor. Substantial infrasound was recorded at distances of hundreds to thousands of kilometres from the vent, beginning at the onset of the eruption and continuing for weeks. Analysis of ground deformation suggests the eruption was fed by a shallow, NW–SE-trending dike, which is consistent with field and satellite observations of vent distributions. Despite lack of prior planning and preparedness for volcanic events in the country, rapid coordination of the emergency response mitigated the human costs of the eruption.

## Introduction

On 12 June 2011, Nabro volcano was the site of an eruption that had significant societal and environmental consequences. It is the first eruption of Nabro on record. Previously, the only securely recorded historical eruption in Eritrea was that of Dubbi volcano in 1861, located just over 30 km from the Red Sea coast (Wiart and Oppenheimer 2000; Wiart et al. 2000). That event reportedly claimed 175 lives despite the sparse population of the Southern Red Sea region of Eritrea, likely as a result of inundation of settled areas by pyroclastic density currents (Wiart and Oppenheimer 2000). Ash clouds and tephra fall associated with the 1861 eruption also disrupted navigation in the Red Sea.

**Fig. 1:**
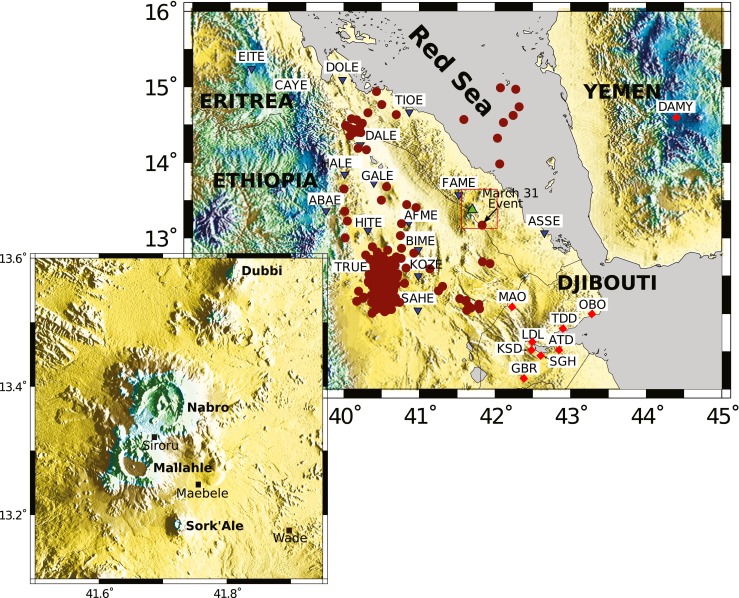
Seismic stations used in our study (*inverted blue triangles* and *red diamonds* indicate temporary and permanent stations, respectively) and regional seismicity (*red dots*) from the USGS catalogue for the period 1960–2011. The *green triangle* locates Nabro. *Inset map* shows the volcanoes in the Bidu Volcanic Complex (coverage of the *red box* in the larger map)

Nabro volcano, some 25 km southwest from Dubbi, is part of a much larger massif referred to as the Bidu Volcanic Complex (Wiart and Oppenheimer 2005; Fig. 1), which is comprised of two calderas (Nabro and, just across the international border in Ethiopia, Mallahle). Nabro’s 2011 eruption began with very little warning—at the time, there were no seismic or other monitoring networks operating in Eritrea. As we show here, seismometers in Ethiopia, Yemen and Djibouti did record seismicity associated with the volcano, but they did not provide information of operational value at the time of the eruption. However, felt earthquakes on the volcano, occurring over several hours before the eruption, did prompt a rapid evacuation of settlements, notably those within Nabro’s caldera. This likely saved many lives. Nevertheless, seven people were killed and three others injured during the eruption. About 12,000 people were ultimately displaced and cared for in temporary camps in the region (Solomon 2012).

In broad terms, Nabro is sited in an extensional setting in the geographical area of Afar, close to the Mesozoic crustal block of the Danakil Alps. It reaches a maximum elevation of over 2,200 m above sea level and has an 8-km-wide summit caldera with associated ignimbrites (Wiart and Oppenheimer 2005). The axis of the Bidu Volcanic Massif, i.e. the alignment of volcanic centres, bears NE–SW, in contrast to the NW–SE trend of the Red Sea and axial volcanic ranges in northern Afar. The Nabro eruption occurred during a period of apparently heightened tectonic and volcanic activity in the Afar/Red Sea region (Yirgu et al. 2014; Jónsson and Xu 2015), which began with the Manda Hararo rifting episode and Dabbahu eruption in Ethiopia (in 2005; e.g. Grandin et al. 2009). Further, there have been eruptions of Dalaffilla (in 2008; Keir et al. 2013) and Erta ‘Ale (2010; Field et al. 2012) on the Erta ‘Ale range in Ethiopia and Jebel al Tair (2007) and Zubair (2011–13) in the southern Red Sea (Xu et al. 2015).

The 2011 Nabro eruption began shortly before midnight local time on 12 June following several hours of ground shaking. The preceding and accompanying seismicity is the first of note in global catalogues for this part of Afar. Remarkably, the eruption produced the largest stratospheric aerosol perturbation since the 1991 Pinatubo eruption (Fromm et al. 2014). This reflected the substantial SO_2_ yield, estimated at around 4.5 Tg for the first 15 days of the eruption by Theys et al. (2013), and penetration of the plume into the lower stratosphere (Fromm et al. 2014; Clarisse et al. 2014). It has been argued that Nabro’s is one of a number of eruptions in the twenty-first century to have had a detectable effect on near-global troposphere temperature, sea surface temperature and precipitation, thereby contributing to the apparent ‘hiatus’ in tropospheric warming since 1998 (Santer et al 2015).

We present here a preliminary synthesis of the nature and mechanisms of the eruption based on multiple observations, obtained via means of satellite remote sensing, regional seismic and infrasound data, fieldwork and petrology. In particular, we aim to reach a first-order characterisation of the eruption in respect of the geometries and kinematics of associated intrusive activity, the compositions of lavas and tephra, the regional context of extensional tectonics and the hazards and humanitarian response. We hope to stimulate further work on the eruption and, more widely, on volcanism in the region. Our study is also relevant to any future work that might consider the geothermal potential of Nabro volcano.

## Methods

We outline here the principal datasets and techniques used in this multi-disciplinary study. In addition to those described below, we have incorporated datasets obtained from operational Ozone Monitoring Instrument (OMI) SO_2_ retrievals (Yang et al. 2007; Sealing 2013) and a Comprehensive Nuclear Test-Ban Treaty Organisation (CTBTO) infrasound station (IS32) in Nairobi, 1,708 km from Nabro (Fee et al. 2013). Additionally, we report in the Overview of the petrology of the 2011 lavas and tephra section a digest of conventional XRF analyses of the whole-rock compositions of samples of lava and tephra erupted at Nabro in 2011.

### Seismology

At the time of the eruption of Nabro in 2011, there were no operational seismometers in Eritrea (triggered permanent stations in the country were out of service) nor was there any routine surveillance of the volcano. The first broadband instruments in the country were deployed (serendipitously) on 23 June 2011, 11 days after the eruption began. This deployment of six sensors was supported by a collaborative project between several of the authors, with the aim of studying regional mantle structure (Hammond et al. 2013). Fortunately, in the months preceding the eruption, seismometers were operating elsewhere in Afar, and we obtained data from ten stations in Ethiopia (Hammond et al. 2013; Barnie et al. 2015b), eight in Djibouti (for which we received arrival time picks) and one in Yemen. Combined, the data from these networks enable us to characterise the seismicity before, during and after the Nabro eruption (Fig. 1). We focus our analysis on events spanning the period from 23 February to 17 September 2011 (3.5 months before the eruption to 3 months after). We note that in August 2011, eight broadband stations were deployed around the volcano (Hamlyn et al. 2014). These provided a valuable 14-month dataset that is especially suited to studies of the relaxation of the system after the eruption. We did not include these data in the present analysis.

We used HYPOINVERSE-2000 (Klein 2002) to locate events from measurements of P- and S-wave arrival times, drawing on a 1D velocity model previously used for investigations in the Afar region (Keir et al. 2009). This model is based on the crustal structure deduced from wide-angle controlled-source seismology (Makris and Ginzburg 1987). We located earthquakes using a minimum of five stations. Typical horizontal errors are <10 km, but depths are only poorly constrained. When we added data from the more proximal Eritrean stations (available from 23 June 2011), errors are reduced, but locations are systematically shifted ∼2 km to the NE. We also relocated events by double-difference methods (Waldhauser and Ellsworth 2000), reducing relative location errors (relative to the master event errors) to between 2 and 50 m, with only six events having relative location errors exceeding 20 m. Local magnitudes were calculated using amplitudes measured on the horizontal components of simulated Wood Anderson displacement seismographs and the distance correction for the Main Ethiopian rift (Keir et al. 2006, 2011). Seismic moment release was estimated using empirical relationships between *M*_L_, *m*_b_ and *M*_o_ (e.g. Kanamori, 1977; Scordilis 2006) as previously applied in a study of Dallol volcano (Nobile et al. 2012). We used Real-time Seismic Amplitude Measurement (RSAM) to follow temporal changes in seismicity (Endo and Murray 1991). This was calculated from average amplitude of a 10-min moving window for the vertical component at selected stations.

### Spaceborne synthetic aperture radar imagery

#### Data

We have analysed spaceborne synthetic aperture radar (SAR) imagery to characterise surface changes associated with the Nabro eruption. We examined backscatter images acquired through time to track the evolution of lava flows and tephra deposits, and of large-scale topographic changes, and to measure surface motion based on interferometric processing and image correlation. Data were acquired from the X-band SAR sensors TerraSAR-X (operated by DLR) and COSMO-SkyMed (operated by ASI). The satellites provide SAR images with nominal spatial resolutions of 2 m for TerraSAR-X (‘Stripmap mode’) and about 1 m for COSMO-SkyMed (‘Spotlight’ mode).

Our differential analyses require identical acquisition geometry for the SAR images, to avoid geometric distortions and artefacts arising from differences in illumination directions. Pairwise combinations are thus restricted to intervals of 11 days for TerraSAR-X and 9 days for COSMO-SkyMed.

#### Mapping volcanic deposits

**Fig. 2:**
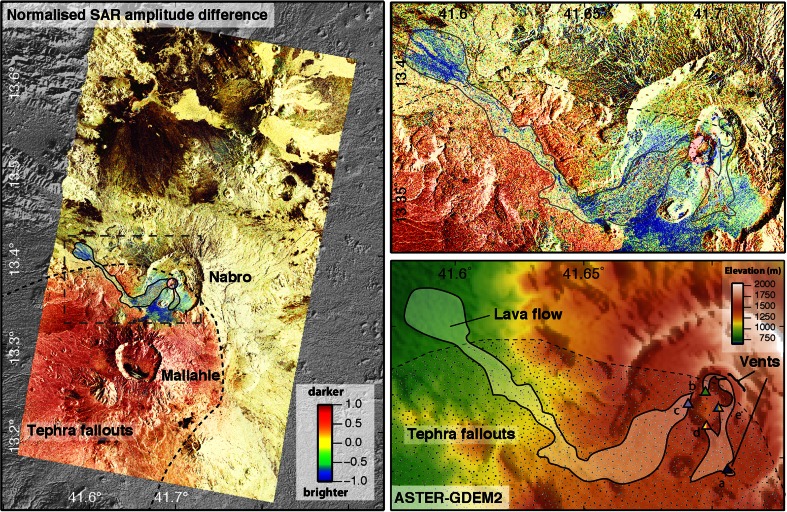
*Left panel* shows a differential analysis of backscattered SAR amplitude for the Nabro area based on analysis of two TerraSAR-X scenes from 11 June 2010 (pre-eruption) and 1 July 2011. The normalised difference of the amplitude images is computed using the formula: Delta_amp = 2 × (amp_1 − amp_2) / (amp_1 + amp_2). *Red areas* indicate decreased SAR amplitude and topographic smoothing (at the cm scale), while *blue areas* correspond to increased SAR amplitude and increased surface roughness. The *upper-right panel* shows the Nabro caldera and 2011 lava flow. The *lower-right panel* shows the corresponding topography. *Coloured triangles* indicate different generations of eruptive vents identified from time series analysis of SAR and thermal imagery (Fig. 10)

X-band SAR images can map geological structures down to the decametric scale. However, geometric distortions associated with complex topography complicate their interpretation. At large scale (from tens of pixels across), the average amplitude of the SAR images is mostly sensitive to the local illumination angle and therefore to slope. Due to the inclined line-of-sight direction of SAR images (usually about 30° from the vertical), systematic geometric distortions and radiometric variations are evident in scenes with topographic relief: while slopes facing towards the sensor appear bright and compressed, slopes inclined away from the satellite are darker and stretched. SAR amplitude images thus provide a qualitative characterisation of topographic changes (Fig. 2).

At the pixel scale, SAR amplitude also depends on backscattering characteristics of the surface. The latter are determined by the dielectric properties of the surface and surface roughness at the scale of the radar wavelength (about 1 cm in our case). We differentiated the SAR amplitude images acquired before and after the eruption to identify the location and nature of deposits (Fig. 2). The newly emplaced lava flows generally appear bright, likely due to high surface roughness. In contrast, decreased backscattered amplitude indicates ‘smoothing’ of the topography by tephra deposits. A significant decrease of the SAR amplitude can be seen in a 20 × 20-km^2^ area to the SW of Nabro.

#### Ground deformation analysis

**Fig. 3:**
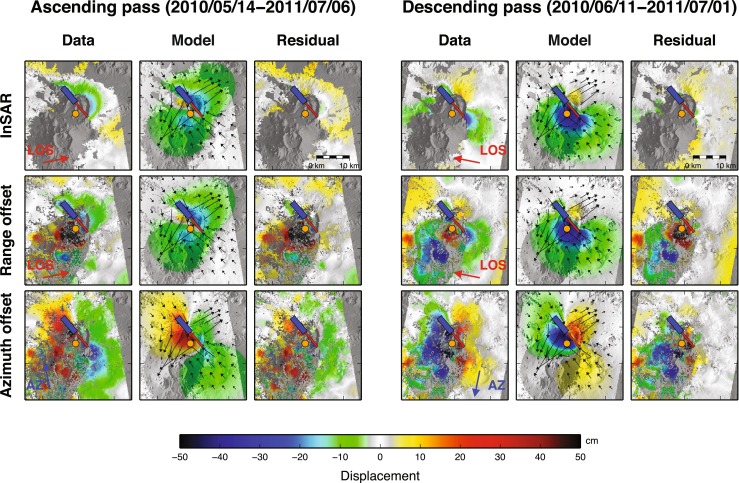
Comparison of observed (*Data*), predicted (*Model*) and residual (*Residual*) deformation for the various components of ground motion deduced from ascending (*left panel*) and descending (*right panel*) SAR data. The *top row* corresponds to InSAR, the *middle row* to range offsets and the *lower row* to azimuth offsets. The *thick-coloured arrows* show the direction of sensed ground displacement: *red* for line-of-sight (LOS) and *blue* for along-track (AZ). InSAR and range offsets are sensitive to motion only in the LOS direction. The LOS vector makes an angle of 29° with the vertical for the ascending pass (34° for the descending pass). For clarity, only the horizontal component of the LOS vector is shown by the *red arrow*. In contrast, azimuth offsets only provide information on the horizontal component of ground motion in the direction of the satellite track (AZ). In each panel, motion in the direction indicated by the *thick-coloured arrow* has negative sign (*cool hues*), whereas motion in the opposite direction has positive sign (*warm hues*). Note the colour scale is common to all components. *Thin arrows* represent the modelled horizontal component of ground motion derived from the elastic inversion. The *shaded area* SW of Nabro received substantial tephra fallout and was excluded in the inversion. The *blue rectangle* represents the surface projection of the normal fault deduced from the model (Fig. 14). The *red rectangle* shows the surface projection of the dike, and the *orange circle* the surface projection of the pressure source. See Fig. 12 for a perspective view

We have analysed ground deformation associated with the eruption in two ways. First, the line-of-sight component of ground displacement is retrieved via interferometric SAR (InSAR) analysis of pairs of SAR images spanning the eruption. We used the NSBAS software, a modified version of ROI_PAC adapted to processing of time series SAR images (Rosen et al. 2004; Doin et al. 2011). The InSAR method requires preservation of surface properties of the ground—it thus fails where tephra deposits or lava flows have accumulated between image pairs. Thus, we only obtain meaningful InSAR measurements beyond Nabro’s caldera rim. InSAR is sensitive only to the component of ground motion that is directed towards or away from the satellite, in the line-of-sight (LOS) direction, about 30° from nadir. Accordingly, we computed interferograms on descending and ascending overpasses (looking from ESE and WSW, respectively) to evaluate the importance of horizontal and vertical components of ground motion (Fig. 3). For SAR data captured after the eruption began, we selected scenes that were acquired within a short time of each other to ensure that the InSAR pairs captured comparable deformation fields. We focus here on two pairs of SAR images acquired by TerraSAR-X: 11 June 2010 and 1 July 2011 for the descending pass and 14 May 2010 and 6 July 2011 for the ascending pass. An analysis of ground deformation spanning the period July 2011 to September 2012 (after the main phase of the eruption) is provided by Hamlyn et al. (2014).

In addition to InSAR, we performed a sub-pixel correlation of SAR amplitude images using the ‘ampcor’ routine of ROI_PAC (Fig. 3). This resolves two components of the ground motion along the native coordinates of the SAR images: horizontal motion along the satellite track (the ‘azimuth offset’) and motion towards or away from the satellite LOS, in the slant direction (the ‘range offset’). Displacements exceeding around 10 % of the pixel size can be resolved with this method, yielding a detection threshold of 10–20 cm, suitable for measuring large deformation (Grandin et al. 2009). The component of displacement sensed by sub-pixel correlation in the slant range direction is the same as for InSAR but does not saturate in zones of large deformation. Furthermore, in contrast to InSAR, sub-pixel correlation can provide meaningful measurements in tephra-covered areas so long as underlying landforms are still apparent. However, it is difficult to distinguish actual motion from apparent motion due to the addition of material at the surface. Such spurious effects are likely to particularly contaminate the range offsets, as the slant range component is nearly vertical. This should only suggest apparent uplift. In contrast, azimuth (horizontal) offsets, when measurable, should be little biased by resurfacing by tephra deposits.

**Fig. 4:**
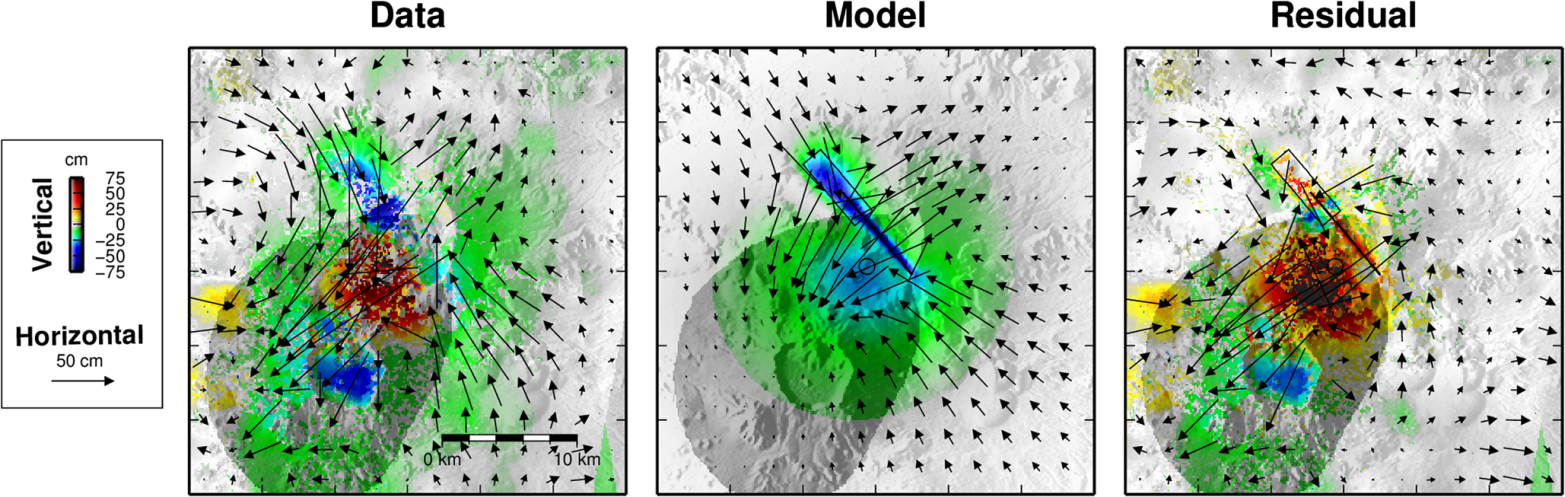
As preceding figure but for ground motion resolved in east–west, north–south and up–down components. The *left panel* corresponds to a reconstruction of the 3D components of ground motion by combining the six available components of ground motion, among which only four yield independent directions. The *colours* represent the vertical displacement, while *arrows* indicate the horizontal component

Finally, using two satellite overpass directions (ascending and descending), four independent components of the ground motion can be resolved (motion in the slant range direction is determined simultaneously by InSAR and sub-pixel correlation). These four independent components permit, after solving an over-determined set of linear equations for each pixel, retrieval of the east–west, north–south and up–down components of ground displacement (Fig. 4).

### Satellite optical imagery

We use satellite images from several sources to track the eruption by its thermal emissions and geomorphologic changes on the ground. These include the following four instruments: the Spinning Enhanced Visible and Infrared Imager (SEVIRI), a multispectral imager aboard Eumetsat’s Meteosat geostationary weather satellites; the Moderate-resolution imaging spectroradiometer (MODIS), a multispectral imager aboard NASA’s Aqua and Terra satellites; the Advanced Very High-Resolution Radiometer (AVHRR), another multispectral imager aboard various NASA and Eumetsat satellites; and the Advanced Land Imager (ALI), aboard NASA’s Earth Observing 1 (EO-1) satellite. SEVIRI has a spatial resolution of about 3 km at nadir, with 96 acquisitions per day; MODIS has a spatial resolution of 1 km at nadir with approximately four acquisitions per day; AVHRR has a spatial resolution of 1 km with a few acquisitions per day; and ALI has a spatial resolution of 30 m with acquisitions typically every few days to weeks when tasked to view an event, either manually or automatically.

We interpret the MODIS, AVHRR and ALI images visually. They contain useful spatial information but are acquired infrequently. In contrast, the SEVIRI images enable near-continuous monitoring albeit at comparatively coarse spatial resolution. We extract the volcanogenic thermal signal from the SEVIRI dataset following Barnie and Oppenheimer (2015a) as follows. Independent component analysis (ICA) is applied to the time series of radiances associated with each non-saturated band 4 (3.9 μm) pixel, in a 9 × 9 pixel widow surrounding the eruption, to find statistically independent contributing sources. The magnitude of the contribution of each source to each pixel is found, again excluding saturated hotspots. Volcanic sources are identified by a high temperature event (HTE) threshold (the product of the absolute skewness of the source and per-pixel contributions). The per-pixel volcanogenic radiances are then found by summing the outer product of the source and contributions for all sources above the threshold. In this way, the image cube is decomposed into a number of radiance sources, the non-volcanic sources excluded, and the image cube reassembled with only non-saturated volcanic sources. For events with many saturated pixels, the correction may underestimate the true radiance but not as substantially as the truncated saturated values. Estimating the contribution of cloud contamination to any given pixel is challenging, and interpretations of the time series are necessarily qualitative: for instance, a drop in radiance could result from either occlusion of hot material or declining lava effusion. We note also that the vent and lava flow were frequently obscured by the volcanic plume.

### Fieldwork

Fieldwork was conducted between 8 and 15 October 2011 by a subset of the authors (BG, CO, JOSH, EY and GK), based first at Afambo and later at Wade. Given the limited time, the extent of the lava flow fields and tephra fall, and the proximity of the international border, a comprehensive sampling strategy was impractical. However, lavas and tephra were sampled over a wide area within and beyond the caldera.

Meaningful estimates of lava flow thicknesses were not possible given the dimensions of the flow field. The flow surfaces were well above ambient temperatures in places and were still emitting fumes. The longest flow, which had travelled beyond the caldera, had overrun cultivated and settled areas. It was sampled towards its front. The vent region was approached from the northern part of the caldera. Within the caldera, three shorter flows were sampled.

A second field mission was conducted in October 2012 to decommission the temporary seismic network. It afforded time for a reconnaissance of the vent region from the southern part of the caldera. The two missions provide some relevant observations but were not focused on obtaining specific ground truth with which to validate interpretations based on our analysis of satellite and other monitoring data.

## The eruption: observations and models

In this section, we report key observations (seismology, satellite remote sensing and ground surveys) and explanatory models for intrusive and eruptive processes involved in the 2011 eruption.

### Seismicity

The area around Nabro has hitherto appeared to be seismically quiet. No major earthquake was reported by international or regional centres (USGS or ISC) prior to the eruption, with the exception of a 4.5-*M*_W_ event on 31 March 2011 (i.e. just over 10 weeks before the eruption). This was the first earthquake ever reported by the USGS for the area (Fig. 1). The epicentral coordinates given by the USGS for this event are 13.17° N, 41.83° E (horizontal error > 20 km), i.e. about 25 km southeast of Nabro, with an origin time of 18:33:38 UTC.

**Fig. 5:**
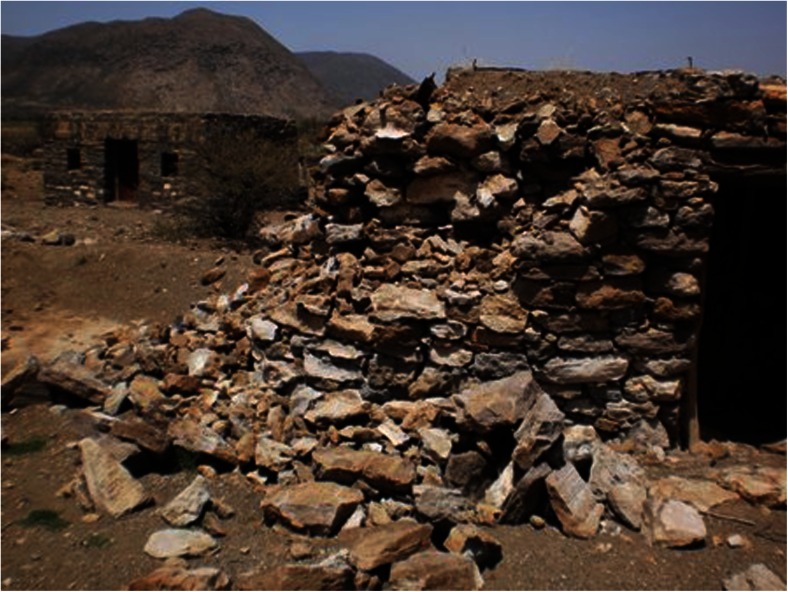
Building damaged by the 31 March 2011 earthquake. Better constructed walls, as seen in the background, sustained only cracking

On 15 May 2011, a scientific team from Asmara visited the area (Ogubazghi et al. 2014) to investigate the effects of this earthquake and to interview eyewitnesses. It was found that most houses within the Nabro caldera were damaged, with the severity ranging from cracked walls to partial collapse (Fig. 5). The event had also induced landslides and, according to local people, killed many domesticated animals including sheep and goats. Small tensional cracks were still present on the ground (Ogubazghi et al. 2014). The length of these cracks reached up to 10 m but, reportedly, they had extended several tens of metres immediately after the event. The cracks trended NNW–SSE, parallel to the direction of plate opening determined from plate kinematic and moment tensor analyses (e.g. McClusky et al. 2010).

**Fig. 6:**
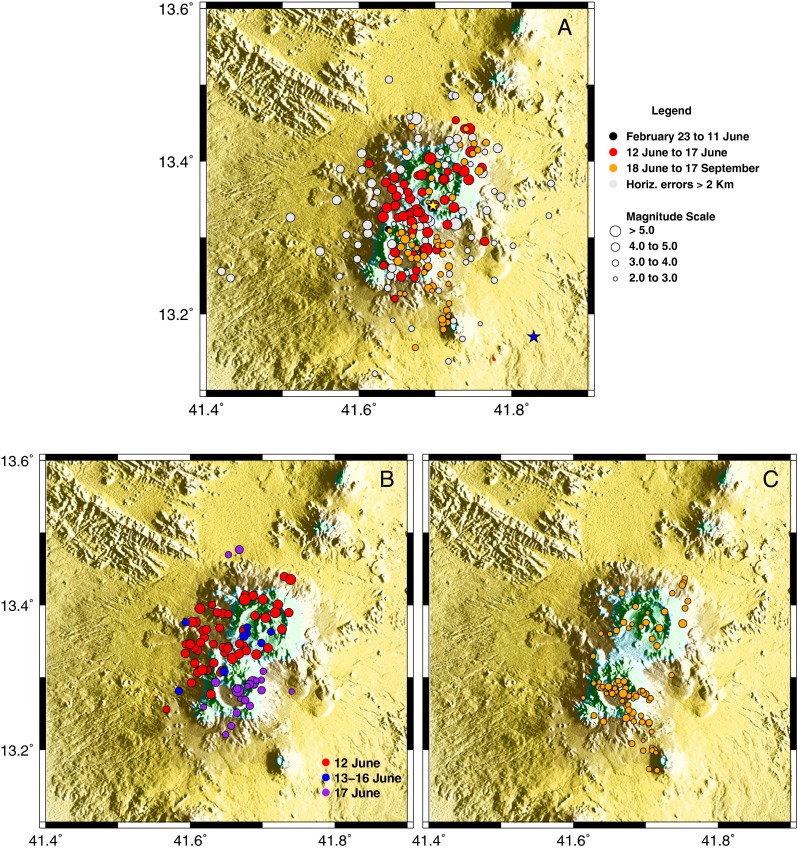
**a** Seismicity around Nabro for the period 23 February to 17 September 2011. Locations for the 31 March event are shown by the *blue star* (USGS location based on distant stations of the Global Seismic Network) and *yellow star* (our location based on stations in Ethiopia and Djibouti). *Colour codes* display temporal evolution, events with horizontal errors exceeding 2 km are *coloured grey* and symbol size represents magnitude (*M*_L_) classes (see legend). Plots (**b**) and (**c**) show double-difference relative locations for events occurring between 12 and 17 June and between 18 June and 17 September 2011, respectively

While residents of Siroru, a village situated on the southwest floor of Nabro’s caldera, clearly felt the shaking, people in the vicinity of Maebele and Wade (close to the USGS-located epicentre) were not awoken by the event. Moreover, damaged houses were observed around Siroru but not in Wade or Maebele. A maximum intensity of VII was estimated for the Siroru area and of less than IV in Wade and Maebele (Ogubazghi et al. 2014). Based on these observations, the epicentre was relocated to 13.33° N, 41.68° E, placing it within the caldera (Ogubazghi et al. 2014). This is close to our relocation of the earthquake, using data from the stations in Ethiopia and Djibouti, to 13.35° N, 41.69° E (horizontal error of ±1.2 km), with a magnitude *M*_L_ 4.8 and time of 18:33:37 UTC on 31 March 2011 (yellow star in Fig. 6a).

From analysis of the regional data, we detected just six other events in the Nabro region before 12 June, with *M*_L_ ranging between 2.8 and 3.9. We did not identify any earthquakes in the days preceding the eruption (the last one was on 29 May) earthquakes with magnitudes below our detection threshold of *M*_L_ ∼ 2.1 may well have occurred.

**Fig. 7:**
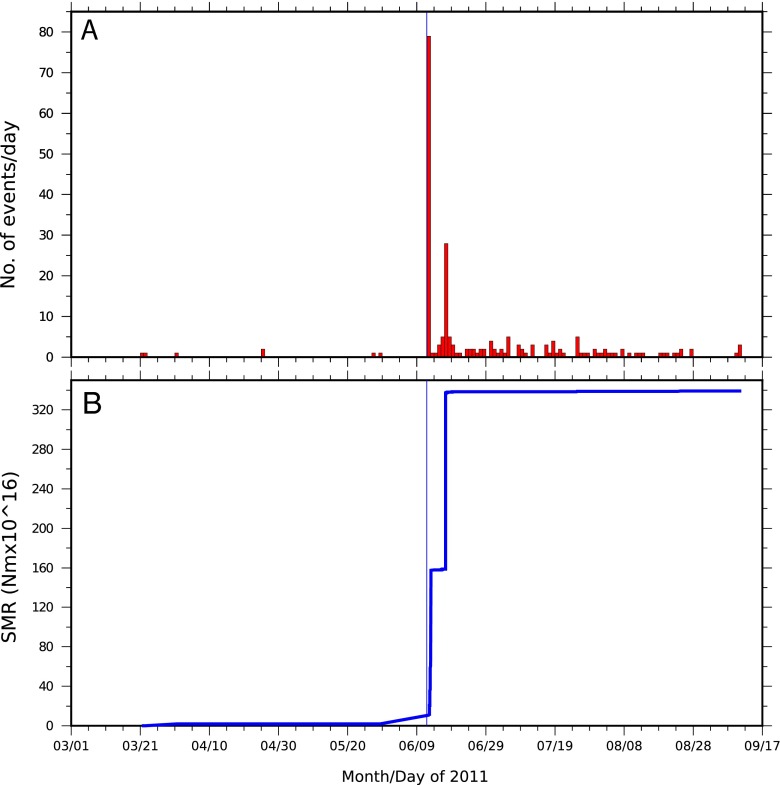
Time series of **a** daily totals of seismic events located using the Ethiopian and Djibouti stations and **b** corresponding seismic moment release (SMR)

The eruption was preceded by 5 h of earthquake swarms, with the first event at 15.37 UTC on 12 June 2011. Two further notable events occurred at 20:32 and 21:03 UTC, the earlier of the two likely very close to the onset of the eruption (Fig. 7).

The most seismically active days were 12 and 17 June, with 79 events (>*M*_L_ 2.0) on 12 June and 28 events on 17 June (totalling 55 % of the recorded events). Local magnitudes during this period vary between *M*_L_ 2.7 to 5.9, with ten events exceeding *M*_L_ 5. The largest event (*M*_L_ 5.9) occurred on 17 June; the second largest, at *M*_L_ 5.8, took place on 12 June. On 12 June, seismicity occurred beneath both Nabro and Mallahle volcanoes. In contrast, on 17 June, while there was little seismicity at Nabro, Mallahle remained seismically active (Fig. 6b). This suggests a linkage between the two volcanoes, either via their magmatic systems or associated with stress field changes. This shift in seismicity to the SW precedes the availability of data from the Eritrean stations, so it is not an artefact of changing station coverage. Mallahle remained seismically active in the months after the eruption, as seen from analysis of data from the local seismic network installed in August 2011 (Hamlyn et al. 2014).

**Fig. 8:**
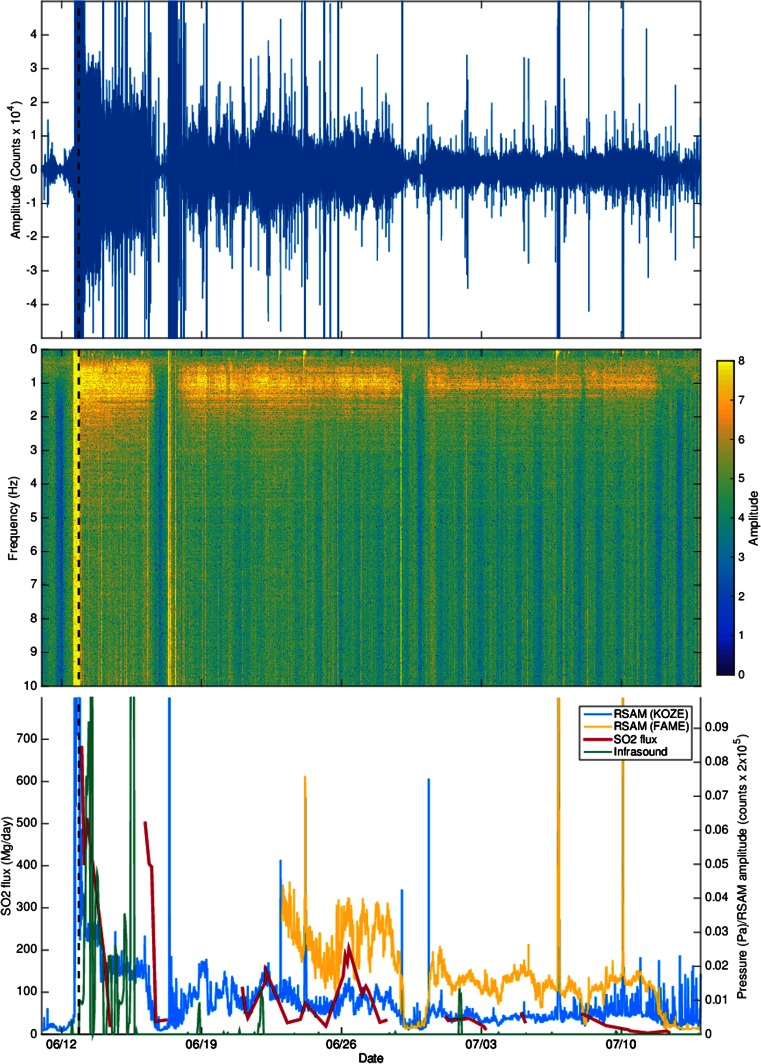
**a** Raw vertical component seismogram from station KOZE, 120 km from Nabro volcano. **b** Spectrogram from station KOZE. **c** Composite plot showing RSAM at stations KOZE (*blue*) and FAME (*yellow*), SO_2_ emission rate computed from OMI data (*red*) and infrasound signal recorded at the Nairobi station (*green*). SO_2_ emission rates were derived using a plume traverse technique (e.g. Theys et al. 2013), assuming SO_2_ advection in a constant wind field, with wind speed derived from a trajectory model. The magnitude and temporal variation of our OMI-based SO_2_ fluxes are broadly consistent with analyses of other satellite SO_2_ measurements for Nabro (Theys et al. 2013). The *dashed vertical line* indicates the eruption onset (20:27–20:42) estimated from SEVERI images

While only ten earthquakes were recorded between 12 and 17 June, RSAM analysis of seismograms from the regional stations indicates sustained tremor (Fig. 8a). Spectrograms for the closest stations, KOZE and FAME, reveal a signal dominated by energy at frequency bands <2 Hz (Fig. 8b). The tremor was very high on the first day of the eruption but reduced during the next 2 days. On 16 June, the tremor almost stopped, but it picked up abruptly with the onset of a second period of intense seismicity on 17 June. After this, tremor persisted until 14 July with many fluctuations (Fig. 8c). The tremor was observed at many stations with different azimuths precluding interpretation of the observed signal as a path effect (e.g. Cote et al. 2010). For the period 18 June to 17 September, we located 93 events (43 % of the total events in our analysis). Events were detected on most days, with 3 days marked by earthquakes sized >*M*_L_ 4.

Looking at the double-difference relative locations, we identify a NE–SW trend (i.e. parallel to the Nabro-Mallahle axis) in epicentres beneath Mallahle on 17 June (Fig. 6b). In contrast, after 17 June, the distribution of epicentres on Mallahle follows more of a NW–SE (Red Sea) trend (stretching as far as Sork ‘Ale volcano; Fig. 6c).

### Observations from spaceborne optical and SAR imagers

The Nabro caldera is up to 8 km across (north to south). In its elevated centre, prior to the 2011 eruption, there were two craters, one about 400 m deep and 2 km across and the other, partly recessed into its SW rim, up to 1.4 km across and over 200 m deep. This smaller crater, whose floor was about 100 m deeper than its neighbour and which we refer to as the SW pit, proved to be the main focus of the 2011 eruption. On its floor were the remnants of a small low-aspect-ratio crater about 140 m across. The inner walls of the SW pit, especially on its east side, were draped with what appear from satellite images to have been scoriaceous lavas, perhaps resulting from drain-back of lava. These lavas are less evident on the western wall of the SW pit, which showed signs of substantial hydrothermal alteration.

**Fig. 9:**
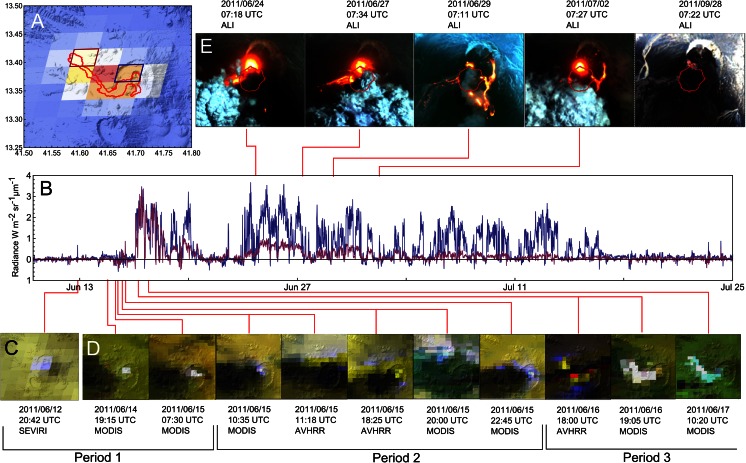
Spaceborne optical observations of the Nabro eruption. **a** A 3.9-μm image of the saturation-corrected volcanic radiance extracted from SEVIRI at 15:12 UTC on 16 June, superimposed on GDEM topography for comparison. Low-to-high radiance is scaled from *blue*-*to*-*red*, and the final extent of the lava flow is outlined in *red*. Pixels covering the vent area and lava flow front are outlined in *blue* and *purple*, respectively. The time series of volcanogenic radiance for these pixels are shown in **b**. Note that the radiance time series are ‘spiky’ due to presence of cloud and plume. **c** The SEVIRI image that signals the start of the eruption (and providing the isolated spike in radiance seen late on 12 June in **b**, before the vent is obscured by plume). **d** Low-resolution images tracking the eruption onset, with bands around 4, 10 and 12 μm as RGB, respectively, in each case. Saturation of AVHRR channels results in loss of a strip of pixels, leading to the banding effect seen in some images. During period 1, the thermal anomaly is restricted to the SW pit in the Nabro caldera, while during period 2, a weak thermal anomaly around the plume margins may result from forward scattering of thermal emission from the advancing lava flow indicating breaching of the SW pit. Period 2 is associated with weak thermal anomalies in the SEVIRI time series seen in **b**. Period 3 follows the first break in the plume, revealing that lava has reached almost its final extent by this point. **e** High-resolution shortwave infrared images of the vent region during the later stages of the eruption. *Red polygon* marks the outline of the original SW pit

The time series of SEVIRI images indicates that the eruption started between 20:27 and 20:42 UTC on 12 June, i.e. shortly before midnight local time as confirmed by eyewitnesses. This is evident in Fig. 9a, b, which shows a measure of thermal radiation for two pixels, one representing the final lava flow front and the other the vent region inside the caldera. The signal is ‘spiky’ due to the intermittent presence of thick plume and cloud that obscure the hot material at the surface. Figure 9c shows the first SEVIRI image to register the eruption, showing an incipient thermal anomaly and plume, placing eruption onset between the time of acquisition of this image and its predecessor (20:42 and 20:27 UTC, respectively). While the SEVIRI images usefully reveal the structure and dispersal of the plume, the thermal anomalies on the ground are obscured until 15 June.

**Fig. 10:**
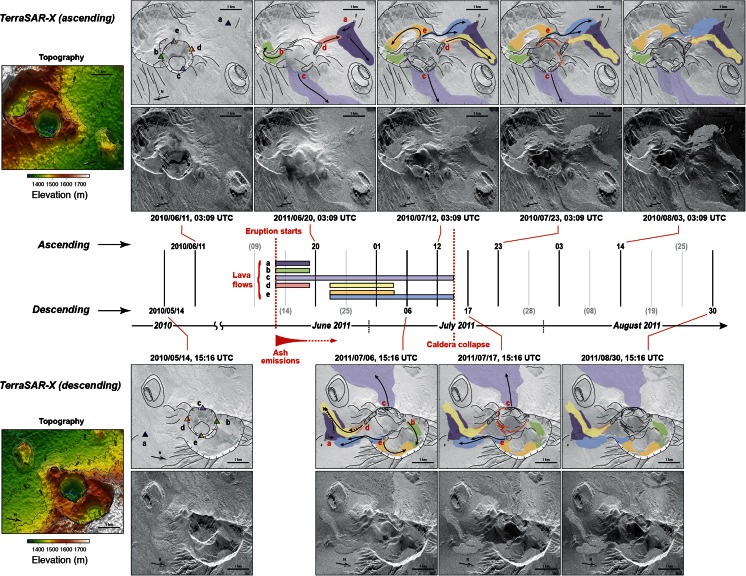
Time series of SAR images focused on the source region of the flows, with interpretations of lava flow emplacement and topographic changes. *Upper and lower panels* show ascending and descending geometry, respectively (in the native geometry of the sensor to avoid artefacts caused by erroneous orthorectification due to syn-eruptive topographic changes; see Fig. 3). The images have been rotated to facilitate interpretation. In each SAR image, the far range is at the *bottom*, and the near range is at the *top*. Note significant distortions and radiometric changes associated with steep slopes. The *central panel* shows lava flow emplacement through time, with the dates of the images used for interpretations (dates indicated in *grey* were unavailable)

The first high-spatial resolution image we have is an ALI scene acquired at 07:32 UTC on 14 June, i.e. about 35 h after the start of the eruption. A plume rich in condensed steam drifts to the west. It covers most of the caldera, hiding the vent area from view. The next spatially detailed scene, a COSMO-SkyMed SAR image acquired on 18 June, 5 days after the eruption onset, reveals substantial changes compared with a pre-eruption scene—(i) the innermost pit crater has been largely infilled by a tephra cone, (ii) several lava flows have issued from points on and around this cone, (iii) the longest of these flows has breached the open SW margin of the caldera and then travelled NW and (iv) a discontinuous alignment of small pits has opened up along a NW–SE trend, reaching about 2 km from the vent region. Inspection of the pre-eruption topography indicates that the main lava flow (labelled (*c*) in Fig. 10) was sourced at the lowest point on the SW pit rim (about 1,515 m above sea level), on its western side.

To follow events of the first days of the eruption, we have to turn to lower-spatial resolution sensors including MODIS and AVHRR. Figure 9d shows thermal images from these instruments covering the period from 14 to 17 June. The interval can be subdivided into three periods. In period 1, the plume dominates the SEVIRI signal, and the MODIS/AVHRR images show a small thermal anomaly confined to the crater. In period 2, the plume margins develop thermal anomalies at 3.9 μm, which could be due to forward scattering of infrared radiation from the advancing lava flow. Minor thermal anomalies are also registered in the lava flow front pixel indicating that it had covered most of the distance by the evening of 15 June. During period 3 (from the evening of 16 June), the plume has weakened, revealing the thermal anomaly at the vent and showing the lava flow has reached its maximum length by this point.

Returning to the high-spatial resolution imagery, we have two more SAR scenes acquired on 20 and 21 June before the next optical images from ALI were recorded (Fig. 9e). Dramatic topographic changes occurred in the vicinity of Nabro’s former SW crater. As a result, images acquired syn- or post-eruption that are geo-coded with a pre-eruption DEM are severely distorted in this area (see Fig. 2). Therefore, we analyse changes in the native geometry of the SAR system. The alternating ascending and descending passes of the satellite further complicate this comparison (successive passes in the same direction are acquired every 11 days by TerraSAR-X). The most prominent change apparent in SAR imagery is the complete filling of the SW crater, already seen in the first image acquired after the eruption onset (20 June). Several new scoria cones are also identified. We calculate that the SW pit was filled from 1,350 m elevation (pre-eruptive crater floor) to at least 1,550 m elevation (highest altitude of overflow of the crater rim), requiring a volume of lava and tephra exceeding 0.1 km^3^.

The ALI scenes acquired between 07:18 UTC on 24 June and 07:27 UTC on 2 July are quite spectacular (Fig. 9e), notably in the shortwave infrared (SWIR) channels. These indicate intense thermal radiation around the vent area located close to the highest point on the SW crater (on its NNE rim) and along a number of lava flows. In all these images, the source of the main lava flow remains ‘hot’ in SWIR channels, though the intensity of the anomaly has diminished by 29 June. The front of this flow appears still active on 24 June. Video taken by one of us (SB) near the flow front reveals widespread exposure of incandescent lava along the flow margins, in addition to flames leaping from engulfed trees. The strong thermal anomaly centred on the new tephra cone is suggestive of a perched lava lake filling a flared vent.

The main lava flow (labelled (*c*) in Fig. 10) initially travelled SW from the vent region until reaching the breached part of the horseshoe-shaped caldera. At this point, it turned NW, reaching an overall flow length of about 17.5 km. In addition, both SAR images and ALI infrared scenes reveal successive generations of smaller lava flows emplaced to the S, E and N of the vent region. With the exception of a short lava flow fed from a series of aligned vents to the SW of the old crater (flow (*a*) in Fig. 10), all these lava flows appear to originate from four distinct sources at the four cardinal points of the SW pit (flows (*b*), (*c*), (*d*), (*e*) in Fig. 10). The first lava flows were sourced at points around the W (*c*), N (*b*) and S ((*a*) and (*d*)) rims of the SW crater. Vents located to the north of the rim ((*b*) and (*e*)) sourced lava flows that were confined to a pre-existing pit crater. Lava supply from vent (*b*) had ceased by 20 June. In contrast, the flows emitted by vent (*d*) were emplaced in two phases: a short flow was emitted before 20 June and was subsequently covered by a longer flow that remained active until at least 1 July. Finally, onset of effusive activity at vent (*e*) seems to have occurred after 20 June and continued until at least 12 July. We note that vent (*c*), which sourced the main lava flow, marked the lowest point of the pre-existing crater rim (1,515 m elevation). Vent (*e*), the second longest-lived, was located at 1,525 m elevation; vent (*d*), the third longest-lived, at 1,545 m elevation; and vent (*b*) stands at an altitude of 1,585 m. This pattern may suggest a progressive lowering of the magma column.

**Fig. 11:**
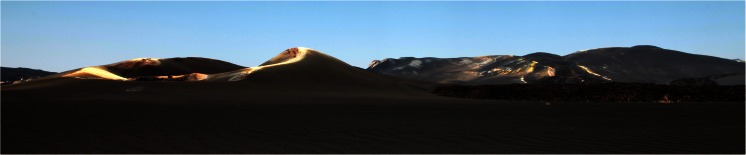
Photograph of part of the fissure system taken in 2012. View approximately to the W—a small cone, formed in the 2011 eruption, is apparent on the left and the front of lava effused from vent (*e*) (Fig. 10) apparent on the right. Behind is the modified cone of the SW pit crater

It is not until 29 June, when there remains only a weak ashy plume drifting to the south, that the ground surface south and southeast of the tephra cone becomes unobscured. A smaller lava flow has advanced east from the SW pit rim and then diverged with one branch heading north where it ponded in the adjacent crater and another moving south down the flank of intracaldera edifice. High temperatures are evident along levées of the main lava flow, where incandescent lava was likely more exposed. By 2 July, the eastern lava flow had advanced further into the adjacent crater. Particularly striking in the 29 June ALI SWIR bands is the revelation that the aligned cinder cones seen in the SAR images from 18 June onwards coincide with intense thermal anomalies. The location and orientation of these vents suggest the possibility of their source being a NW–SE-striking dike (Fig. 11). These vents do not appear to have contributed significantly to lava eruption after 20 June.

Notably, one of these extends from the SE rim of the SW crater, which appears to have been substantially infilled by lava and tephra. The main flow appears to be at its maximum extent, though the ‘spillway’ at the vent that sources it remains ‘hot’, suggesting sustained lava effusion, in the shortwave infrared channels.

Comparing the SAR images acquired on 18 June and 4 July indicates that the main flow only advanced a further 400–500 m in the intervening period, reaching 750 m elevation, and a total length of approximately 17.5 km. The ALI images show that between 24 June and 2 July, the main lava flow advanced only ∼140 m at its front, though it likely continued to thicken and widen in places along its length during this period. Between 17 and 23 July, the SAR images reveal drainage of lava from the main flow’s channel, quite close to the source and within the caldera. There has been a minor advance of the intra-caldera flow to the SE. As early as 20 June, scarps are apparent around the tephra cone, where the original rim of the pit crater is being expressed or exposed. Much more of the new tephra cone apparently collapsed later in July, though between 23 July and 8 August, no further substantive changes are evident in the SAR imagery.

By 28 September, the SWIR channels of the ALI image indicate anomalous radiation only in association with hot spots around the vent region and along the rim of the original pit crater. The lava flows appear ‘cold’ at these wavelengths. This pattern persists in the 6 October ALI scene but, 10 days later, the anomalies around the vent region in the SWIR bands look less intense. This is consistent with observations made by several of us during fieldwork carried out on the volcano between 8 and 15 October. We observed incandescent vents in the vent area from the caldera rim on the night of 12 October and a weak plume drifting across the caldera during the daytime.

A prominent signature of the initial flows emitted from vents (*a*) and (*c*) (Fig. 10) is that their texture appears to be smoother in the first image acquired after the start of the eruption (20 June) than seen subsequently. The smooth texture of early lava flows seen in the SAR images could reflect lower surface roughnesses of the lavas or, more likely, tephra fallout from the early phases of the eruption (the effects of which are readily apparent elsewhere within the caldera; Fig. 2). This would suggest that the output of ash diminished substantially after 20 June, as later lava flows retain their ‘blocky’ appearance thereafter.

In the SAR images acquired after 20 June, we see that lava eruption from sources (*d*), (*c*) and (*e*) (Fig. 10) continued up to early to mid-July. This is consistent with the volcanic thermal radiance registered in the pixel situated above the vent, which persisted until around midday on 16 July, before decaying rapidly to low values before midnight (Fig. 9b), marking the cessation of intense eruptive activity. The radiant signal from the flow front decayed significantly by early July, suggesting waning advance. Compared with the 12 July SAR image, the 17 July scene reveals a set of ring faults or fractures associated with subsidence of the newly emplaced central cone. The geometries of these fault traces recapitulate the trace of the former SW pit rim, suggesting localisation of brittle deformation along the interface between the previous crater walls and the new infill of tephra and lava. Lava eruption seems to have ceased in conjunction with this substantial collapse of the material, although minor changes prevailed near vent (*c*), possibly arising from drainage of lava already within the channel or slope instability.

Both the SAR and optical images clearly reveal the fallout from the eruption, fanning out in a 130° sector from the vent area (spanning SSE to WNW). In the SAR images, this is apparent from a decrease in backscattered intensity (area in red in Fig. 2). This reflects a smoothing of the topography at the scale of the radar wavelength (about 1 cm) and decrease of the energy backscattered towards the antenna. The most significant change in radar intensity occurred before the first acquisition (20 June), which suggests that the bulk of the ash was deposited in the first 6 days of the eruption. The area of tephra fallout evident in the SAR imagery covers an area of about 700 km^2^. It is not possible to assess the thickness of tephra on the ground solely from the available satellite images.

### Satellite geodesy

The decomposition of ground displacement into horizontal (east–west and north–south) and vertical components derived from SAR imagery reveals a complex pattern of ground motion (Fig. 4). At the large scale, the horizontal displacement field indicates an overall NE–SW extension, concurrent with NW–SE compression. Maximum horizontal displacements reach 0.75–1 m beyond Nabro’s caldera rim. The vertical component of ground displacement indicates widespread subsidence, reaching an average of 0.2 m to the N, E and SE of Nabro. Localised subsidence, reaching up to 0.5 m, is apparent to the NW of the caldera rim within an area elongated along a NW–SE axis. Displacements retrieved within the caldera and to the SW of the vent region are particularly complex. The most prominent feature is localised uplift reaching about 0.75 m within a circular area, 5 km in diameter. The vents lie on the edge of this zone. Further south, apparent subsidence of Mallahle caldera exceeds 0.5 m. Horizontal displacements are directed to the SW and exceeded 1 m. This area of complex deformation coincides with a decorrelation of the InSAR data (Fig. 4) and a decrease of the backscattered SAR amplitude (Fig. 2). This suggests that thick tephra deposits cover the area and that the retrieved ground motion is at least partly an artefact. We therefore neglect this area in the quantitative analysis of ground deformation that follows.

For a first-order interpretation of the deformation source, we performed a series of forward elastic models to reproduce the main features of the ground displacement field in well-resolved sectors of the volcano and its environs. These were based on models from Okada (1985) and Mogi (1958) to predict surface deformation induced by rectangular dislocations or isotropic point sources, respectively. We tested various combinations of faults, dikes and pressure sources. We found three elementary sources sufficient to explain the pattern and amplitude of ground deformation. Our preferred model includes a NW–SE dike, responsible for the NE–SW horizontal dilation pattern observed to the NE of the volcano. This is consistent with the orientation of vents observed within the crater (Observations from spaceborne optical and SAR imagers section). A negative pressure source beneath the vent area explains the overall subsidence signal, as well as NW–SE contraction. The net NE–SW dilation results from the predominance of dike-induced dilation over Mogi-induced contraction in the direction perpendicular to the dike. In contrast, the dike does not produce significant horizontal motion beyond its tips, so that the effect of the negative pressure source accounts for the NW–SE-oriented pattern of contraction observed to the SE and to the NW of the volcano. Finally, a shallow NW–SE-striking, NE-dipping normal fault reproduces the localised subsidence seen to the NW of Nabro.

**Fig. 12:**
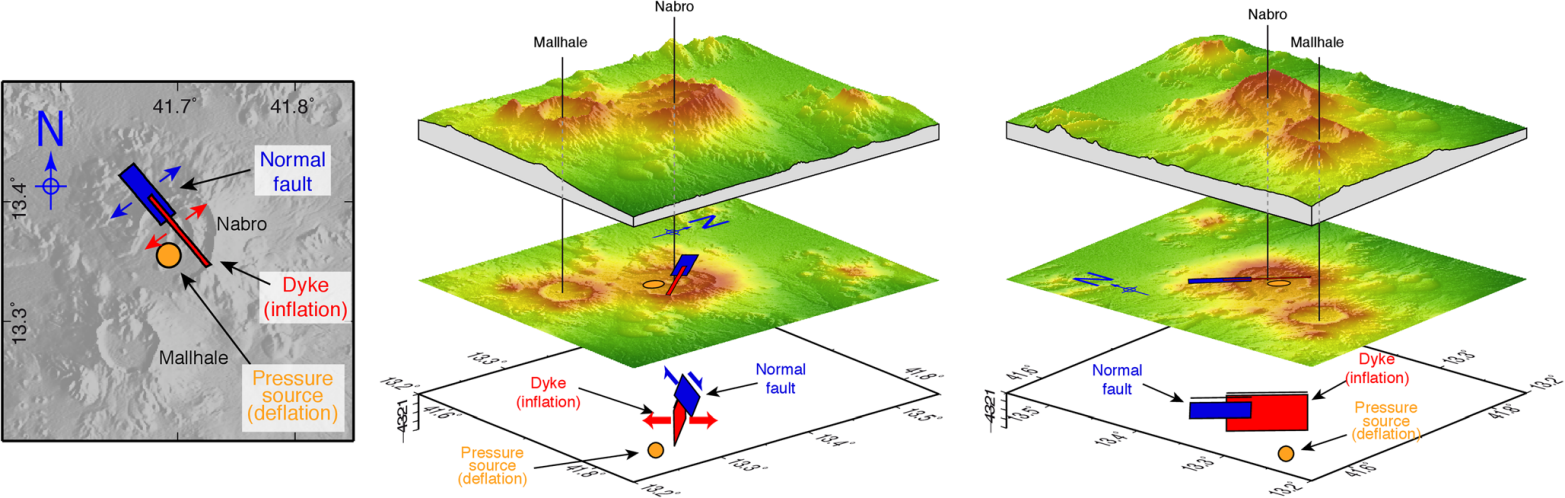
Model deduced from elastic inversion of the SAR-derived surface displacement field. The *left panel* shows the surface projection of the three elementary sources used in the model in map view. The *centre and right panels* show the same sources in a 3D perspective view, looking from the SE and SW, respectively

We then used these three sources of deformation to initialise a nonlinear inversion of the surface deformation that takes the six displacement fields as an input (InSAR, SAR range offset and SAR azimuth offset, for ascending and descending passes). We followed Tarantola and Valette (1982) to optimise the geometry (location, size, dip, depth) and kinematics (slip magnitude and direction or magnitude of pressure change) of the three elementary sources. Not all the parameters could be constrained due to incomplete spatial coverage of the data and various tradeoffs. The best-fit model is shown in perspective view in Fig. 12. This preferred solution includes a negative pressure source equivalent to a volume loss of 0.07 km^3^, situated at 6 km depth. This depth coincides with the depth of a deflating pressure source deduced from analysis of ground deformation derived from InSAR time series (spanning 1 July 2011 to 5 October 2012), and with a cluster of micro-earthquake sources 5 km below sea level, located using the local seismic network operating from 31 August 2011 (Hamlyn et al. 2014). The modelled dike is vertical, strikes at 320° N, is 8-km-long and extends from 0.3 km down to 4.3 km depth. It corresponds to a volume inflation of 0.04 km^3^, which is comparable to the volume lost from the underlying pressure source. As noted, the strike direction of the dike is roughly parallel to the alignment of the vents identified from high-resolution imagery (Observations from spaceborne optical and SAR imagers section). This trend is perpendicular to the direction of regional spreading associated with motion of the Arabian and Nubian plates.

**Fig. 13:**
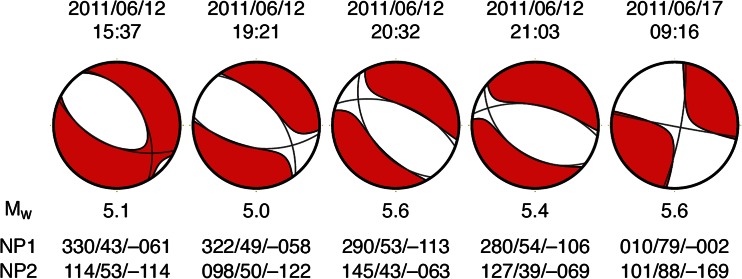
Moment tensors for all events with moment magnitudes (*M*_w_) exceeding 5.0 and detected near Nabro during June 2011 and recorded in the Global Centroid Moment Tensor (gCMT) catalogue (Dziewonski et al. 1981; Ekström and G.M. and Dziewonski, A.M. 2012). The plots show the full moment tensor of each event, with *grey lines* showing the fault planes of the double-couple component of the moment tensor. Strike, dip and rake of the two nodal planes are indicated

Lastly, the normal fault strikes at 320° N, slips by 0.7 m, has a dip angle of 55° and extends from 0.8 km down to 3.2 km depth. The seismic moment associated with this fault is 4.3 × 10^17^ N m, which is equivalent to an *M*_w_ 5.8 earthquake (assuming a shear modulus of 3.3 × 10^10^ Pa). This is comparable to the sum of the two most energetic earthquakes that occurred on the day of peak seismic activity on 12 June, at 20:32 and 21:03 UTC, with magnitudes *M*_w_ 5.6 and *M*_w_ 5.4, respectively (2.91 × 10^17^ and 1.42 × 10^17^ N m according to gCMT). The focal mechanisms of these two earthquakes indicate normal faulting striking 280–290° N, albeit with a substantial non-double-couple component (Fig. 13). Given the uncertainties in the analysis, this is broadly consistent with the 320° N direction determined from our interferometric SAR analysis.

**Fig. 14:**
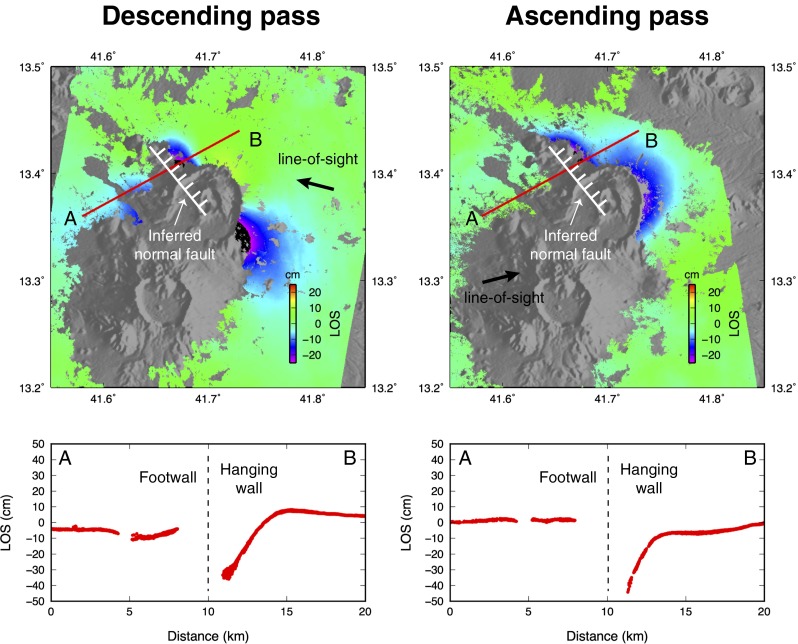
Descending (*left*) and ascending (*right*) interferograms showing the line-of-sight component of ground displacement around Nabro volcano. *Blue-to-purple* shading corresponds to motion away from the satellite. The *thick black arrows* show the line-of-sight direction. The *red lines* indicate the profiles across an inferred normal fault (surface trace in *white with bars* indicating the hanging wall), shown in the corresponding *lower panels*

Overall, the elastic modelling suggests that the Nabro eruption was associated with a shallow dike intrusion. This dike may have linked a deeper source to the surface eruption. The onset of seismicity suggests the dike intruded on 12 June. It triggered slip on one or several normal faults running parallel to the dike at shallower depth, as observed elsewhere in regions undergoing local or regional extension (e.g. Rubin and Pollard 1988; Rowland et al. 2007). It is evident from our InSAR analysis that faulting breached the surface on the NW flank of Nabro. Figure 14 shows the co-eruptive displacement along a profile perpendicular to the inferred normal fault. Motion away from the satellite to the NE of the surface trace of the fault clearly points towards a normal fault dipping to the NE with most of the vertical displacement concentrated in the hanging wall. Fault geometry in the vent region cannot be readily identified due to resurfacing by lava flows and tephra deposits.

Dike intrusions are common during volcanic eruptions. However, the broad scale of fringes in the interferograms indicates that the dike opened permanently with some magma retained in the crust (i.e. not all was erupted). This inflation of the dike suggests a response to the regional extensional stress field and a degree of tectonic influence on the eruption. However, the relative importance of tectonics versus magmatic processes in nucleating, feeding and maintaining the inferred dike remains unclear. It is interesting that the initial dike injection beneath Nabro followed a NW–SE, Red Sea trend (Satellite geodesy section), while on 17 June, the seismicity shifted to Mallahle and defined a NE–SW trend (Fig. 6b). Seismicity beneath Mallahle then switched back to more of a NW–SE, Red Sea trend, stretching as far as Sork ‘Ale volcano (Fig. 6c). This may indicate a perturbation of the stress regime in which the direction of maximum horizontal stress changed. A closer study of earthquake source mechanisms and seismic anisotropy may provide further evidence and insights.

The residual displacement field determined after subtracting the best-fit model reveals several other notable features. Beyond the masked area (shaded zones in Figs. 3 and 4), the residual horizontal and vertical displacements are generally small. In contrast, a residual uplift, reaching about 1 m, is seen to the SW of the vent region. This is likely the result of tephra accumulation in the first days of the eruption. A distinct negative anomaly, corresponding to subsidence, is also apparent within the caldera of Mallahle volcano. This residual signal might correspond to deflation of a reservoir below Mallahle, induced by activity of the neighbouring magmatic system. This activation of Mallahle is seen also in the seismicity, beginning 17 June (Seismicity section) and continuing in late 2011 (Hamlyn et al. 2014).

While our model captures the main features of the ground deformation field, we emphasise that it is non-unique for several reasons: (i) the retrieved surface displacements do not span exactly the same observation intervals, (ii) spatial coverage of the data is patchy due to the tephra fall deposits, (iii) sub-pixel correlation results are fairly noisy around the volcano, likely due to slope effects and relatively adverse surface conditions for X-band SAR imaging (compared with the Afar plains), (iv) the deformation field is particularly complex and (v) the elastic inversion problem is inherently non-unique. Thus, alternative solutions based on a different set of assumptions could be proposed. For instance, Hamiel et al. (2013) explained the first-order contribution to the observed deformation as a NNE–SSW-trending, left lateral, strike-slip fault located at shallow depth beneath Nabro volcano, consistent with the focal mechanism of one of the most energetic earthquakes of the eruption episode (09:16 UTC, 17 June 2011, *M*_w_ 5.6).

### Synthesis of observations

Figure 8c integrates observations of the Nairobi infrasound station, RSAM and SO_2_ emission rates computed from available OMI satellite data. Several features stand out in the time series: (i) the persistence of seismic tremor and its infrasonic equivalent (i.e. continuous vibration of the atmosphere) during and after the main eruptive stage, (ii) approximate correlations between the temporal trends in the three parameters, (iii) a pronounced drop in RSAM to near zero around 17 June and (iv) plateau-like temporal signals in RSAM prior to 17 June but more fluctuating trends afterwards. Most of the located events occurred on 12 and 17 June. The largest earthquakes also occurred on these days.

The SEVIRI images bracket the eruption onset to between approximately 20:27 and 20:42 UTC on 12 June—the later scene shows a pronounced thermal spike over the caldera. The prominent earthquake at 20:32 UTC could signify the final dike advance that breaks the surface. The infrasound recorded at the closest station in Djibouti (264 km from Nabro) picks up abruptly at 21:44 UTC (Fee et al. 2013). For an acoustic travel time of circa 20 min from Nabro, this indicates that explosive activity either began or picked up substantially at 21:24 UTC. This may indicate that the first 50 min or so of the eruption saw predominantly effusive activity at one or more vents, although the SEVIRI images indicate a plume that rapidly gains altitude between 20:42 and 20:57 UTC.

Optical and SAR observations indicate that the main lava flow had advanced to close to its maximum extent within about 3 days of the eruption onset on 12 June. In addition, most of the tephra was emplaced within about 6 days of the onset. The persistent tremor between 12 and 16 June suggests sustained high lava effusion rates during this period. On the other hand, infrasonic signals point to continuous but fluctuating explosive activity, consistent with the evidence for airborne ash and tephra accumulation on the ground. Unique features in the infrasound signal suggest the volcanic jet was gas-rich and supersonic (Fee et al. 2013). SO_2_ emissions were strong during this period, likely with efficient decoupling of gas from erupting magma at the vent(s). The comparative quiescence in activity around 16/17 June is puzzling but may correspond to a choking in magma supply to the vent(s).

The abrupt resumption of tremor on 17 June and accompanying seismicity suggests renewed magma supply. Interestingly, most of the seismicity is located beneath Mallahle volcano, suggesting either triggering by changes in the local stress field (e.g. Walter et al. 2014) or potentially interconnection between magma reservoirs (e.g. Albino and Sigmundsson 2014). The former explanation seems more likely based on the observation that post-eruption seismicity (31 August–7 October 2011) beneath Mallahle had significantly lower *b* values than that associated with Nabro (Hamlyn et al. 2014). Optical imagery indicates vigorous activity at the vent region, likely characterised by a lava lake sourcing a steam-rich, low-altitude plume. SO_2_ was still detected by OMI but burdens and emission rates were reduced, suggesting lower lava effusion rates and consistent with generally low explosivity at the vent(s). Explosive activity on 18 and 21/22 June is apparent in the Nairobi infrasound data and coincides with a pulse in RSAM and SO_2_ output. By 29 June, the optical shortwave infrared images (ALI) indicate diminished lava effusion. While SO_2_ emissions persisted, outputs were further reduced.

### Overview of the petrology of the 2011 lavas and tephra

**Fig. 15:**
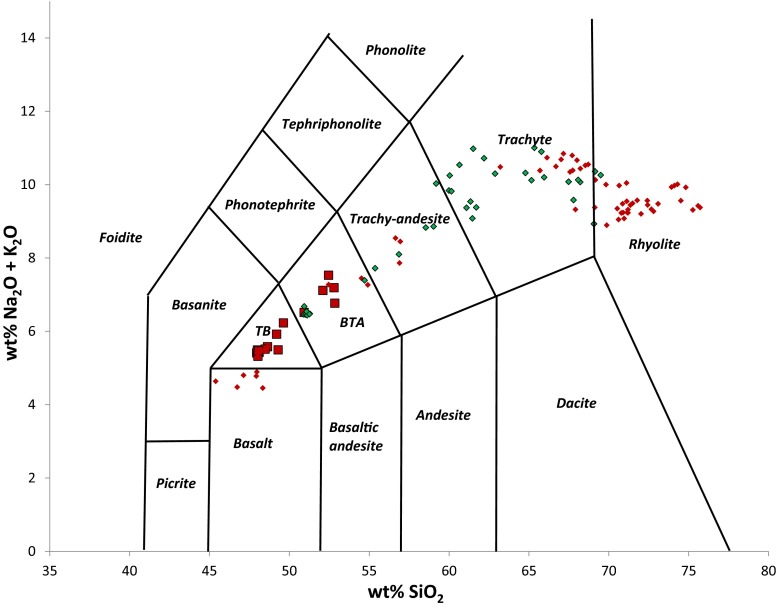
Total alkalis versus silica plot for eruptive products from the 2011 eruption

The lava erupted is trachybasaltic to trachybasaltic andesite in composition—between 48–53 wt% SiO_2_ and 5–7 wt% total alkalis, following a mildly alkali-enriched trend relative to rocks from the axial volcanic ranges in Afar (e.g. Barberi et al. 1972; Fig. 15). Whole-rock MgO contents are low (3–5 wt%). There are abundant phenocrysts of olivine, clinopyroxene, plagioclase and magnetite, with rare ilmenite, chromite and fluorapatite. Microlites of olivine, clinopyroxene, plagioclase and Fe-Ti oxides are present in the ground mass. Some tephra samples are glassy with abundant plagioclase microlites, while others are more crystalline. Phenocrysts show both normal and reverse zoning, indicating a complex plumbing system. This is rare in Afar and may be the result of the presence of thicker crust in the proximity of the Danakil Alps.

Olivine phenocrysts are typically compositionally zoned with fayalite-rich rims and forsteritic cores (Fo about 85). Some contain inclusions of chromite. There are also a few smaller crystals that are reverse-zoned. Clinopyroxenes exhibit diverse textural features. One population has no discernible zoning, while the majority of phenocrysts are normally zoned. A few, however, show slight reverse zoning. Plagioclase is abundant and shows normal zoning, with cores about An_80_ and rims about An_55_. Magnetite and ilmenite are both present and are relatively homogeneous. There are two apatite populations, one containing very little sulphur and the other exhibiting some zoning in sulphur.

The lava is highly crystalline and shows evidence of along-flow fractionation. The variation in the tephra textures may represent discrete batches of magma from depth. There is localised contamination from lithics of rhyolitic composition, most probably introduced in the upper regions of the conduit. Textural and compositional features suggest that some of the magma was reheated prior to the eruption, while other phenocrysts appear to have been transported rapidly from depth. It is likely that there was at least one recharge event prior to the 2011 eruption, which would be consistent with seismic data.

Clinopyroxene-hosted melt inclusions contain up to ∼3,100 ppm S, whereas matrix glass contains very low sulphur. A separate sulphide phase is present in some samples and as inclusions in phenocrysts. It contains up to 3 % Cu and 1.3 % Ni. Taking the SO_2_ mass released into the atmosphere as 4.5 Tg (Theys et al. 2013), a crystallinity of 60 % and the aforementioned value as indicative of melt sulphur content, the dense rock volume of the eruption amounts to approximately 0.47 km^3^ (magnitude 5.1, given by [log_10_ (mass in kg) − 7]). This is roughly compatible with the scale of emplaced lava flows (approximately 18 km^2^ in area for an assumed mean thickness of 20 m) and tephra deposits (5 m mean thickness inside the caldera, 0.1 m mean thickness beyond). We hope in the future to be able to obtain further tandem SAR data products to enable a more accurate determination of the lava and tephra volumes. This will be essential for a robust treatment of the volatile budget.

## Impacts of the eruption and emergency response

The Danakil region is generally dry and sparsely populated. However, Nabro, which rises to more than 2,200 m above sea level, engenders a microclimate that favours denser settlement. Several villages are, or were, located within the caldera and on the flanks of the volcano. Siroru, the main settlement within the caldera, had a population of around 3,000 prior to the eruption. The inhabitants of the region are mostly pastoralists keeping herds of sheep, goats, camels, donkeys and cattle. Despite the limited number of recorded earthquakes, the seismic hazard in the region had been previously recognised (e.g. Midzi et al. 1999). The eruption of Dubbi in 1861 (Wiart and Oppenheimer 2000) is also a testament to the volcanic hazard and is remembered by communities living in the area today. Nevertheless, prior to the 2011 eruption, a national risk mitigation strategy for geophysical threats was lacking. There was no monitoring of any kind on Nabro nor was there a formalised warning system in place.

We believe that the experience of the 4.5 *M*_W_ event on 31 March 2011 may have served to sensitise and increase preparedness of the population in advance of the eruption. Almost all the houses in the affected area that were built with non-mortared stone walls suffered severe damage or collapse; those built with mortared walls suffered varying degrees of cracking (Fig. 5). During the visit of the scientific team in May 2011, village and district administrators and community leaders were briefed on what to do in the event of future earthquakes, namely to vacate their homes and avoid structures that could collapse and areas susceptible to landslides.

Village and district administrators in Eritrea are mostly war veterans well-versed in aspects of emergency management and in coping with situations with limited means at their disposal. Their experience was likely decisive in the immediate local management of the unfolding emergency on 12 June 2011. An army detachment based close to the local administration building in Siroru maintained radio contact with the Southern Red Sea Region Administration (SRSRA) in the regional capital, Assab (about 120 km away, as the crow flies). Together with other nearby units, they helped to evacuate people as the crisis developed on the night of 12/13 June 2011. Clearly, people living within the caldera, on the volcano flanks and in range of the tephra fallout, were most affected by the eruption and its associated seismicity. In particular, Siroru was destroyed by a combination of seismic shaking, lava flows and burial in tephra. While some people refused to be evacuated, by and large the rescue efforts were conducted with the full cooperation and consent of the affected people.

Another factor that may have contributed to the rapid response is that one of us (JOSH) happened to be in Asmara at the time of the eruption. He received an automated e-mail via the USGS National Earthquake Information Center’s ‘Preliminary Determination of Epicenters’ service concerning the first larger earthquake on 12 June (15:33:12 UTC, i.e. 5 h before the eruption). This information was relayed via another member of our team (GO) to the SRSRA.

The first meeting of the SRSRA committee tasked with coordinating the response to the unfolding humanitarian crisis took place at 08:00 local time on 13 June 2011 in Assab. This resulted in the dispatch of a medical team and provision of emergency relief (food supplies and shelters, Solomon, 2012). Other government agencies provided longer term assistance to displaced people in respect of health care and schooling.

The SRSRA classified the displaced persons into two categories. Those in category 1 came from villages that were destroyed. Those in category 2 were those whose villages were threatened by the eruption including the effects of fumes and tephra fallout. According to Solomon (2012), during the first 2 days of the eruption, the estimated numbers in category 1 were 700–800 families (about 2,500–3,000 individuals). The corresponding values for category 2 were about 2,250 families (about 9,000 individuals). On 17 June 2011, when the eruption intensified, most category 2 evacuees were reclassified as category 1. In total, about 11,780 people were affected. Seven people were killed and three were injured during the eruption (Solomon 2012).

The losses in household goods were substantial (Solomon, 2012). In previously settled areas of the caldera floor south of the vent region, tephra accumulated to depths of several metres, burying or partially burying many structures. Two villages were completely destroyed, and recorded losses include 19,839 goats and sheep, 460 cattle, 834 camels and 142 donkeys. Total economic losses were estimated to be in the region of US$3 million (Solomon 2012). The contamination of water wells in the area, at least on the Eritrean side, appears to have been limited (Solomon 2012).

Many of the people displaced by the eruption were unable to return to their homelands; substantial numbers have been permanently resettled elsewhere. Resettlement can have major consequences for livelihoods, communities and culture and can expose people to new hazards (Usamah and Haynes 2012). Resettlement programmes are arguably most effective when the affected communities are closely involved in decision-making. According to Solomon (2012), this appears to have been the case following the Nabro eruption.

Information on impacts on the Ethiopian side of the border is limited, but the caldera of Mallahle volcano was inhabited and received significant fallout of tephra from the eruption plumes. We were told that the affected communities received assistance in Eritrea (although the two countries do not maintain diplomatic relations). Communications received from staff at a mining operation in Dallol, 170 km to the northwest in Ethiopian Danakil (and below sea level) suggested grounding of a portion of the gas and aerosol emissions, causing discomfort to workers. Respiratory stress may have been exacerbated by the extreme temperatures experienced in this part of northern Afar in the summer.

In terms of civil aviation issues, the region falls under the responsibility of the Toulouse Volcanic Ash Advisory Centre. The volcanic clouds resulted in disruptions to aviation in the region with international flights in East Africa and the Middle East particularly affected. In the first days of the eruption, there was confusion in the outside world as to whether it was Dubbi or Nabro that was erupting.

## Concluding remarks and perspectives

The 2011 Nabro eruption offers a valuable opportunity to develop our understanding of unrest and eruptive activity of caldera systems, the local interactions between tectonics and volcanism and between neighbouring volcanoes and the origins and significance of the off-axis volcanic ranges in the wider Afar region. We summarise key observations and preliminary interpretations as follows:

1. The June eruption was preceded by a damaging earthquake (the 31 March 2011 event) and further felt seismicity and earthquake swarms up to 5 h beforehand. (We heard anecdotally from a local guide that the vigour of steam vents inside the caldera had increased in advance of the eruption. These vents were apparently used by people living within the caldera for therapeutic purposes but had heated up to the point where they were no longer comfortable.) These signs indicate that the eruption was presaged by sensible phenomena.
2. The eruption began between 20:27 and 20:42 UTC, according to satellite images, and possibly close to 20:32 UTC, the time of a large earthquake. It may have been predominantly effusive for the first 50 min or so, then sufficiently explosive to be recorded at distant infrasound stations. A 17.5-km-long lava flow was observed on 16 June 2011, exceeding 1 km in width in places. Explosive activity generated significant tephra clouds and a substantial quantity of SO_2_ was released to the atmosphere. Compositions of erupted lavas and tephra range from trachybasalt to basaltic trachyandesite. (At present, the eruption magnitude is poorly constrained, but it could be calculated in future if additional SAR data are acquired for construction of pre- and post-eruption digital topography.)
3. Approximately 12,000 people were displaced as a result of the eruption, and seven fatalities were recorded. Considering that more than 3,000 people lived within the Nabro caldera itself, the toll is remarkably low. This reflects a rapid, largely spontaneous evacuation of settlements close to the vent region, prompted by premonitory ground shaking and the first signs of eruption.
4. Our analysis of regional seismic records for the period 23 February–17 September 2011 revealed 217 events within a 25–40 km radius of Nabro. Seven of these events occurred before the eruption. Their local magnitudes range between 2.1 and 5.9, with 76 events exceeding *M*_L_ 4 and eight events larger than *M*_L_ 5. While the located events identify temporal variation of seismicity beneath Nabro volcano in the lead up to the eruption, we note that our detection threshold is quite high, about *M*_L_ 2.1. Double-difference relocations of the events reveal clustering beneath both Nabro and Mallahle volcanoes and hint at alignments of epicentres, along a NE–SW trend, notably on 17 June 2011 (parallel to the Nabro-Mallahle axis), and, subsequently, along a NW–SE trend (parallel to the Red Sea).
5. Geodetic modelling suggests that the eruption was associated with intrusion of a shallow dike that triggered slip on parallel normal faults. The orientation of this dike is roughly NW–SE, parallel to the trend of an eruptive fissure formed during the eruption and to the Red Sea.
6. In general terms, temporal trends in recorded seismicity, SO_2_ output and infrasound signals are correlated, with several pulses and fluctuations. The onset of the eruption was marked by high seismicity and infrasound followed by strong SO_2_ emissions to the atmosphere. Seismic and infrasonic tremor persisted for about a month. A lull occurred on 16 June, but seismicity increased abruptly on 17 June.

Our study of the Nabro eruption has highlighted the importance of diverse Earth observation techniques for monitoring volcanoes in comparatively remote regions, especially where local ground-based sensor networks are limited or lacking. It also exemplifies the precarious status of risk management where it is not underpinned by operational monitoring.
